# Caesarean Scar Pregnancy: A Case Report

**DOI:** 10.15388/Amed.2022.29.1.17

**Published:** 2022-06-29

**Authors:** Vilius Rudaitis, Gailė Maldutytė, Jūratė Brazauskienė, Mykolas Pavlauskas, Dileta Valančienė

**Affiliations:** Clinic of Obstetrics and Gynaecology, Institute of Clinical Medicine, Faculty of Medicine, Vilnius University, Vilnius, Lithuania; Department of Gynaecology, Center of Obstetrics and Gynaecology, Vilnius University Hospital Santaros Clinics, Vilnius, Lithuania; Faculty of Medicine, Vilnius University, Vilnius, Lithuania; Department of Gynaecology, Center of Obstetrics and Gynaecology, Vilnius University Hospital Santaros Clinics, Vilnius, Lithuania; Department of Gynaecology, Center of Obstetrics and Gynaecology, Vilnius University Hospital Santaros Clinics, Vilnius, Lithuania; Faculty of Medicine, Vilnius University, Vilnius, Lithuania; Radiology and Nuclear Medicine Center, Vilnius University Hospital Santaros Clinics, Vilnius, Lithuania; Department of Radiology, Nuclear Medicine and Medical Physis, Institute of Biomedical Sciences, Faculty of Medicine, Vilnius University, Vilnius, Lithuania; Radiology and Nuclear Medicine Center, Vilnius University Hospital Santaros Clinics, Vilnius, Lithuania

**Keywords:** caesarean section, ectopic pregnancy, ultrasonography, gynaecologic surgical procedures

## Abstract

Caesarean scar pregnancy is a potentially life-threatening gynaecological condition, becoming more common due to steadily increasing rate of caesarean sections worldwide. More than one-third of women presenting with caesarean scar pregnancy are asymptomatic, but over the time if left untreated this condition can lead to the uterine rupture and massive maternal haemorrhage. Therefore it is necessary to diagnose and manage caesarean scar pregnancies properly at the beginning of the first trimester. We present the case of woman with three previous caesarean sections, who was diagnosed with complicated caesarean scar pregnancy and then successfully managed using surgical intervention.

## Santrauka.

Cezario rando nėštumas yra pavojinga ginekologinė patologija, jos dažnis nuolat didėja dėl visame pasaulyje vis dažniau atliekamų cezario pjūvio operacijų. Nors trečdaliui moterų iš pradžių nebūna jokių simptomų, tačiau negydoma ši patologija gali komplikuotis gimdos plyšimu ir gausiu kraujavimu. Dėl šios priežasties ypač svarbu anksti diagnozuoti ir pradėti gydyti cezario rando nėštumą nedelsiant. Straipsnyje aprašomas komplikuoto cezario rando nėštumo, kurį pavyko sėkmingai išgydyti chirurginiu būdu, klinikinis atvejis.

## Introduction

Caesarean scar pregnancy (CSP) is defined by implantation of the trophoblast into the niche of previous caesarean scar site [1, 2, 3]. The prevalence of this condition directly depends on caesarean section rate, which has significantly increased during the last decades and now reaches 21.1 percent worldwide [4]. Although the incidence of CSP range is documented to be as high as 1:1688 of overall pregnancies [5], the CSP remains underdiagnosed and underreported [5, 6]. At the beginning of the first trimester CSP can only be found accidently using ultrasonography in completely asymptomatic women, however in more complicated cases CSP can manifest as severe haemorrhage, acute lower abdominal pain or even collapse due to haemorrhagic shock [7]. The treatment objectives focus on the prevention of these life-threatening complications and the preservation of fertility, where possible [1]. The surgical management together with caesarean scar repair is both safe and effective, moreover it helps to preserve fertility and can reduce the recurrence of CSP [8-10].

## Case report

A 44-year-old asymptomatic woman, gravida 4, para 3, at 7 weeks of gestation presented to the emergency department of Vilnius University Hospital Santaros Clinics for suspected CSP. Past obstetric history included three caesarean sections, the last one performed 10 years ago.

**Fig. 1.**
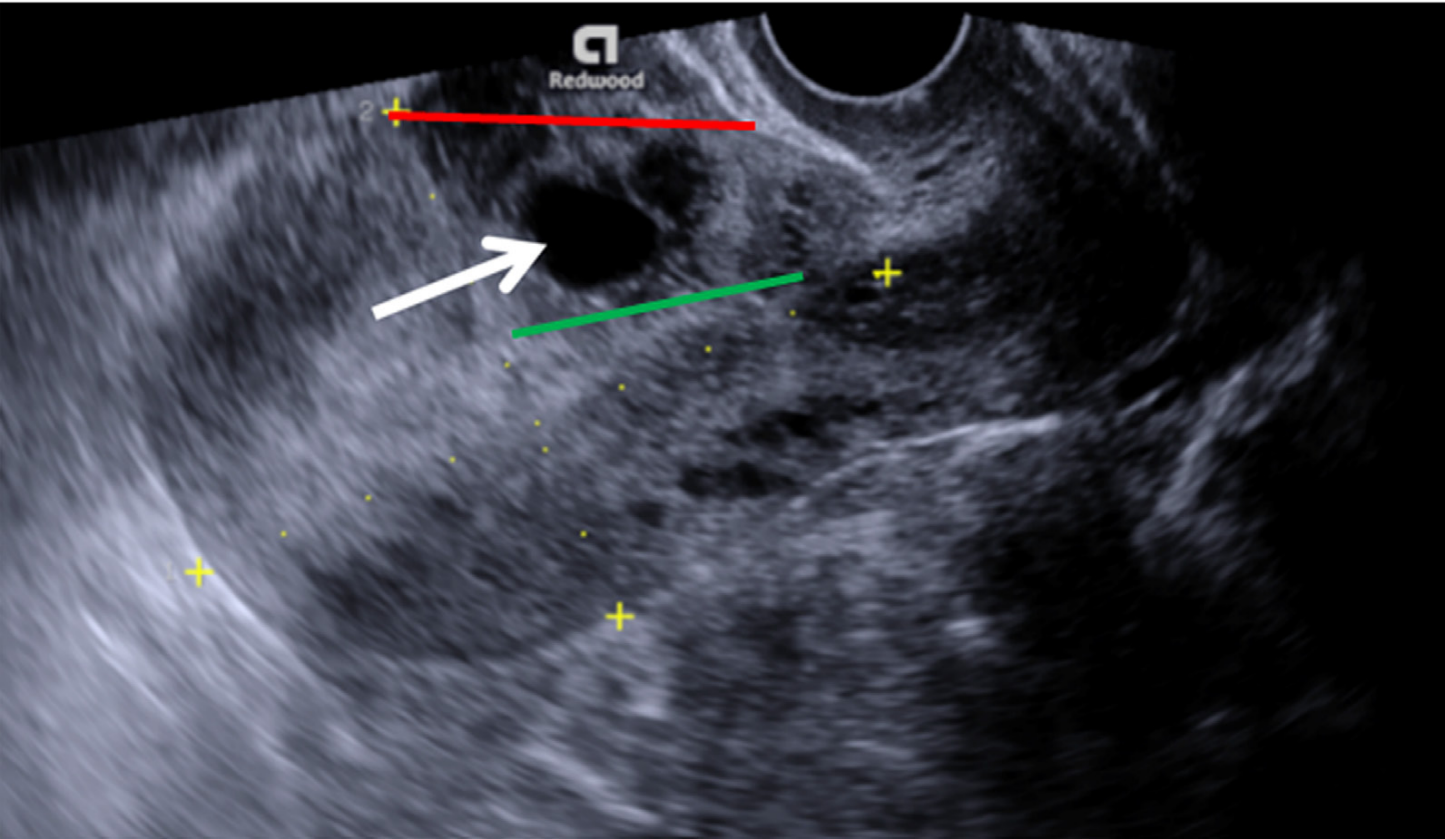
Transvaginal grayscale ultrasound image of the uterus in sagittal plane demonstrates gestational sac (arrow) implanted in the niche of previous caesarean scar site, crossing serosal line (red line), while uterine cavity line (green line) remains intact

On inspection she was found hemodynamically stable with absence of vaginal bleeding. Human chorionic gonadotropin was not measured. Following transvaginal grayscale ultrasonography, irregular gestational sac with the mean diameter of 17 mm without foetal pole and yolk sac was noticed, located in the niche of previous caesarean scar site. Endometrium measured 12 mm, both the uterine cavity and cervical canal were empty, without direct contact to the sac (Fig. 1). The residual myometrial thickness measured 3 mm with the slight protrusion of gestational sac towards urinary bladder. Both adnexa were normal and no free fluid in the pouch of Douglas was found. Colour Doppler imaging demonstrated intense vascularity surrounding the gestational sac. The nonviable CSP was confirmed and expectant management was chosen, while maintaining patient’s wish to preserve her fertility.

**Fig. 2.**
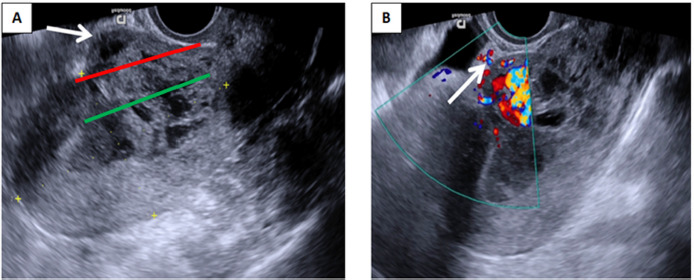
(A) Transvaginal grayscale ultrasound demonstrates heterogeneous mass inside the uterine cavity protruding anteriorly through the scar tissue (arrow). Both the serosal line (red line) and uterine cavity line (green line) are crossed. (B) Colour Doppler imaging reveals intense vascularity in the vesicouterine space (arrow), involving posterior wall of urinary bladder

**Fig. 3.**
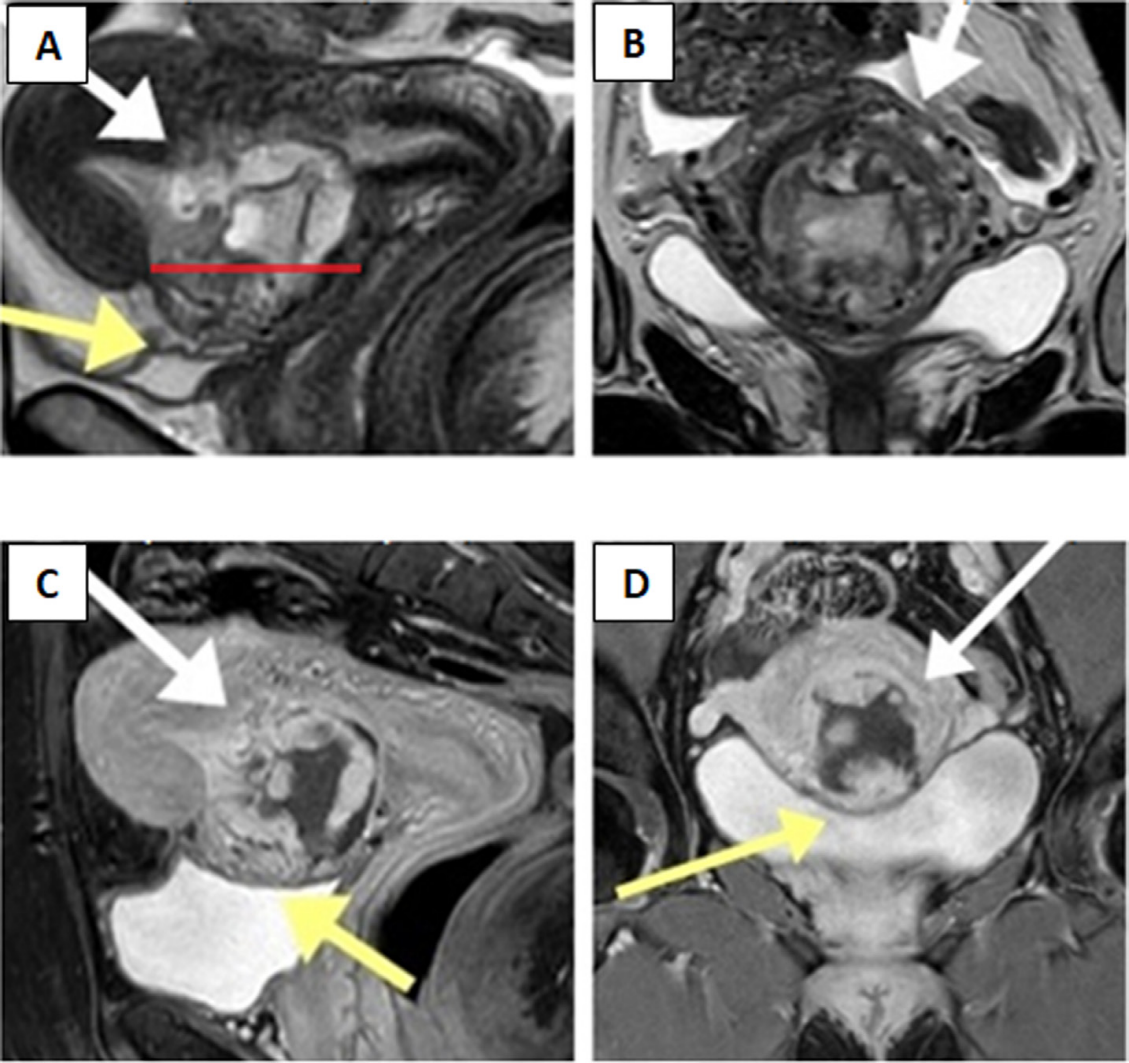
(A) Sagittal T2-weighted magnetic resonance image demonstrates gestational sac as heterogeneous mass in the anterior part of lower uterine wall within the prior caesarean scar site, extending into the uterine cavity (white arrow). The scar margins are separated (red line) following infiltration by ectopic tissue. The “tenting sign’’ (yellow arrow) suggests trophoblast invasion into the wall of urinary bladder. (B) Coronal T2-weighted sequence demonstrates the disruption of thin myometrium secondary to pathological infiltration (white arrows). (C, D) Sagittal and coronal T1-weighted delayed post-contrast magnetic resonance images with full urinary bladder show no “tenting sign’’ (yellow arrows) evident in A image, therefore transmural trophoblast growth into the urinary bladder can be excluded

**Fig. 4.**
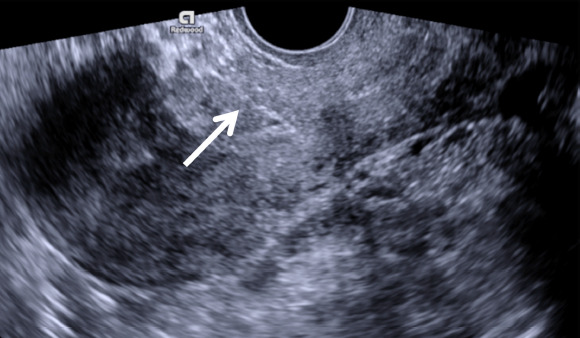
Transvaginal grayscale ultrasound image of the uterus in sagittal plane one month after the surgery demonstrates completely restored caesarean scar site (arrow)

At three weeks follow-up visit patient reported seven days history of vaginal spotting. Following examination, bloody vaginal discharge was observed. Human chorionic gonadotropin was 515 U/l. Transvaginal grayscale ultrasonography showed 55 × 43 mm heterogeneous mass inside the uterine cavity, more extensively protruding anteriorly through the previous caesarean scar. Using colour Doppler imaging it was noticed, that the peritrophoblastic perfusion remained intense, and new hypervascular area in the vesicouterine space was found (Fig. 2). The trophoblast invasion into the posterior wall of urinary bladder was suspected. The magnetic resonance imaging was performed (Fig. 3), however this diagnostic modality did not provide any additional information.

The clinical findings revealed suspicious myometrial integrity disruption and involvement of the posterior wall of urinary bladder, therefore the surgical management was chosen. Patient was admitted to the Gynaecology department for the elective laparotomy. During the surgery intact uterine serosa with the bulging thin uterine wall at the previous caesarean scar site was noticed, no adjacent organ involvement was found. Wedge resection of the lesion with removal of retained products of conception was performed, followed by caesarean scar repair. The recovery was uneventful and the patient was discharged from hospital three days after the surgery. At one month follow-up visit human chorionic gonadotropin concentration decreased to normal level and transvaginal ultrasonography showed completely restored myometrium of 10 mm in thickness at caesarean scar site (Fig. 4). The patient was advised not to conceive for at least six months in order to ensure complete uterine scar healing.

## Discussion

CSP is the trophoblast implantation into the defect caused by impaired healing of the previous caesarean scar [1, 7, 11]. The most common clinical presentation of CSP is vaginal bleeding, which may be anything from spotting to life-threatening haemorrhage, and mild to moderate abdominal pain, while around 37 percent of patients at the beginning of first trimester may be asymptomatic [7]. The most dangerous complication of CSP is uterine rupture, which may present as acute severe pain, massive bleeding and haemoperitoneum, resulting in collapse due to haemodynamic shock [6, 11].

Transvaginal grayscale and colour Doppler combined ultrasonography is the optimal diagnostic modality for the evaluation of suspected CSP [6, 7, 11]. The diagnostic criteria of CPS include I) an empty uterine cavity and endocervical canal, II) a gestational sac or trophoblast located in the niche of previous caesarean scar site, III) a gestational sac with or without foetal pole in the presence or absence of cardiac activity, IV) a thin or absent layer of myometrium between the gestational sac and the urinary bladder and V) an intense trophoblastic or placental perfusion on colour Doppler imaging [1, 2, 6, 11]. According to gestational sac relation with uterine cavity and serosa CSP is classified into three types: I) CSP implanted into the niche with largest part of gestational sac extending into uterine cavity; II) CSP located within myometrium not crossing serosal and uterine cavity lines; III) CSP protruded towards urinary bladder, while crossing serosal line [3]. Magnetic resonance imaging is known to be used as an adjunct to ultrasonography in CSP cases [6, 12]. However, there is no evidence to suggest that magnetic resonance imaging could have any additional value in early CSP diagnosis and should not be used as routine diagnostic modality in CSP cases [3, 6].

It is preferable for the diagnosis of CSP to be made before 9 weeks of gestation, because of significant distinction between CSP, cervical pregnancy and low intrauterine pregnancy at this time [6, 13, 14, 15]. The CSP also has to be differentiated from spontaneous abortion in progress, because the cessation of embryonic or foetal cardiac activity does not eliminate the complications associated with CSP [12]. In our case, the CSP was diagnosed at 7 weeks of gestation, meeting all ultrasonographic criteria and therefore excluding the diagnosis of low intrauterine pregnancy. The cervical pregnancy was ruled out in the absence of its characteristic ultrasonographic features, such as barrel-shaped cervix or the gestational sac location below level of the internal cervical os [1, 11]. Although in our case no foetal pole with embryonic cardiac activity was noticed, the peritrophoblastic perfusion remained intense during all observation periods, therefore the spontaneous abortion was excluded.

Management of CSP depends on clinical presentation, gestational age, CSP location, foetal viability and patient’s wish to continue the pregnancy [6]. Expectant management in viable CSP can be chosen, when patient decides on continuing the pregnancy [2, 5, 6, 14]. In such cases CSP can progress to viable intrauterine pregnancy, while highly increasing the risk of severe maternal morbidity, associated with massive maternal haemorrhage during delivery, caesarean hysterectomy or even death [2, 5, 6]. Therefore if patient consents to terminate the pregnancy, medical or surgical management has to be initiated as early, as CSP diagnosis is confirmed in order to prevent life-threatening maternal complications [2]. Medical management in CSP cases includes systemic and local injection of methotrexate. Local injection of methotrexate into the gestational sac under ultrasound guidance appears to be more effective approach, comparing to systemic intramuscular methotrexate administration, which is no longer recommended as stand-alone treatment option because of increased risk of complications [2, 6, 16]. However, medical management require close monitoring of the patient for several months until gestational mass resolves [6]. The surgical management consists of hysteroscopic resection, vacuum aspiration and excision of CSP with scar reconstruction [2]. Hysteroscopic resection and ultrasound-guided vacuum aspiration are less invasive but very effective surgical methods frequently used to evacuate the pregnancy in uncomplicated CSP cases [2, 6, 10]. The excision of CSP via laparotomy, laparoscopy or transvaginal approach is another surgical treatment option, which enables for the scar defect to be repaired at the time of CSP removal [2, 6, 8, 10]. The resection of the old scar with a new uterine closure can also reduce recurrence of CSP in the future [8-10]. In our case, at the beginning the patient was diagnosed with early nonviable CSP, located within myometrium with the slight protrusion of gestational sac towards urinary bladder (Fig. 1), meeting type III criteria of CSP classification. An expectant management was chosen while waiting for spontaneous abortion to occur. It was the wrong decision because expectant management is not an appropriate option in nonviable CSP, especially in type II and type III cases, because of the higher complications rate. In our case an expectant management resulted in complicated CSP and at three weeks follow-up visit the defect at caesarean scar site and trophoblast involvement of the posterior urinary bladder wall were suspected and the reconstruction of the defect in caesarean scar site was required. The excision of CSP via laparotomy with the repair of uterine wall defect was performed, but during surgery no suspected myometrial integrity disruption was found.

Although the risk of recurrent CSP varies between 5 to 15.6 percent in the subsequent pregnancy, successful intrauterine pregnancies have been reported after the conservative and surgical treatment of previous CSP [17, 18]. In subsequent pregnancy, caesarean section is recommended between 34 0/7 to 35 6/7 weeks of gestation, before the onset of labor [5].

## Conclusions

CSP is a potentially life-threatening gynaecological condition, which has to be diagnosed and treated early at the first trimester. Expectant management is not an appropriate treatment option in nonviable CSP, located completely in the uterine wall or even protruding towards the urinary bladder, because of higher complications rate and greater need for second-line surgical treatment.
